# Comparative efficacy of VMP vs. Rd in newly diagnosed, autologous stem cell transplant-ineligible multiple myeloma patients: a prematurely terminated randomized controlled study, CAREMM-2002 study

**DOI:** 10.1007/s44313-024-00025-7

**Published:** 2024-07-17

**Authors:** Cheong Yoon Huh, Sung-Soo Park, Jung Yeon Lee, Chang-Ki Min, the Catholic Research Network for Multiple Myeloma (CAREMM-2002 study), Young-Woo Jeon, Seung-Ah Yahng, Seung-Hwan Shin

**Affiliations:** Department of Hematology, College of Medicine, Catholic Hematology Hospital, Seoul St. Mary’s Hospital, The Catholic University of Korea, Seoul, Republic of Korea

To the editor

The introduction of novel therapeutics has led to significant advancements in the treatment of transplant-ineligible multiple myeloma (MM) [1]. Based on the results of the VISTA [2] and FIRST [3] trials, bortezomib-melphalan-prednisolone (VMP) and continuous lenalidomide-dexamethasone (Rd) regimens have emerged as standard treatment options for transplant-ineligible MM [4]. However, prospective studies that compare the effectiveness of VMP and Rd, which use distinct drug classes as their foundation, remain lacking. To bridge this gap, our research aimed to prospectively evaluate VMP versus Rd. This study presents the available data from a prematurely concluded comparative study of VMP and Rd.

The study was designed as a randomized controlled, open-label, multicenter trial. Patients were recruited from four university hospitals within the Catholic Research Network for Multiple Myeloma. A total sample size of 552 patients (276 per treatment group) was targeted. Eligible patients were ≥ 18 years old and had newly diagnosed, documented, measurable MM as defined by International Myeloma Working Group (IMWG) criteria [5]. Confirmation was required by one of the following: serum monoclonal protein ≥ 1 g/dL in IgG MM or ≥ 0.5 g/dL in IgA, IgD, IgE, IgM MM; 24-h urine monoclonal protein ≥ 200 mg; or, if monoclonal protein was undetected in serum or urine, involved free light chain (FLC) ≥ 10 mg/dL with an abnormal kappa to lambda FLC ratio. Additional inclusion criteria encompassed an Eastern Cooperative Oncology Group (ECOG) performance score of 0 to 3 and ineligibility for high-dose chemotherapy and autologous stem cell transplantation (ASCT) due to age (≥ 70 years) or comorbidities. Exclusion criteria were primary amyloidosis; polyneuropathy, organomegaly, endocrinopathy, monoclonal protein, and skin changes (POEMS) syndrome; monoclonal gammopathy of undetermined significance; smoldering MM; Waldenström’s macroglobulinemia; or other conditions where IgM monoclonal protein is present without clonal plasma cell infiltration and lytic bone lesions. Also excluded were patients with prior or current systemic therapy for MM, except for emergency use of corticosteroids (equivalent to dexamethasone 40 mg/day for up to 4 days); peripheral neuropathy or neuropathic pain of grade 2 or higher as defined by the National Cancer Institute Common Terminology Criteria for Adverse Events (NCI CTCAE) version 5.0; or active but untreated hepatitis B or C virus or human immunodeficiency virus.

Patient enrollment commenced in May 2020, with screening conducted within 21 days prior to randomization. Using a computerized method, patients were then randomly assigned in a 1:1 ratio to receive either VMP or Rd. In the VMP group, patients received nine 42-day cycles of bortezomib (1.3 mg/m^2^ subcutaneously twice weekly on weeks 1, 2, 4, and 5 of cycle 1 and once weekly on weeks 1, 2, 4, and 5 of cycles 2–9), melphalan (9 mg/m^2^ orally once daily on days 1–4 of each cycle; adjusted to 4.5 mg/m^2^ in patients with baseline serum creatinine > 2 mg/dL), and prednisone (60 mg/m^2^ orally or intravenously once daily on days 1–4 of each cycle). Patients in the Rd group received continuous 28-day cycles of lenalidomide (25 mg once daily on days 1–21 of each cycle) and dexamethasone (40 mg once weekly, adjusted to 20 mg for patients aged > 75 years) until disease progression or intolerance was observed. The Institutional Review Board of each participating hospital approved the study protocol (XC20M IDV0001), which was registered in the Clinical Research Information System of South Korea as #KCT0005006 (registered on May 11, 2020; details available at https://cris.nih.go.kr/cris).

The primary endpoint of the study was overall survival (OS), defined as the duration from randomization to the date of death from any cause or the last follow-up date. The key secondary endpoints included overall response rate (ORR; proportion of patients who achieved a partial response or better), progression-free survival (PFS; time from the date of randomization to either progressive disease or death, whichever occurred first), and safety assessment. The definitions of endpoints, methods for patient evaluation, and methods for statistical analysis are described in Online Resource 1.

Patient enrollment for this study concluded in February 2022, with 27 patients registered during the enrollment period. Of these, 14 and 13 patients were randomly assigned to the VMP and Rd treatment groups, respectively. The median patient age was 71 years (range, 65–81 years). Baseline characteristics were evenly distributed between the two groups (Table 1). High-risk cytogenetic profiles—defined as t(4;14), t(14;16), or del17p—were present in 2 (18.2%) and 6 (46.2%) patients in the VMP and Rd groups, respectively (*p* = 0.202).

**Table 1:** Baseline Characteristics of the Patients

Variables	VMP	Rd	*p*-value
	**(*N* = 14)**	**(*N* = 13)**	
**Age**			
Median (range)	72 (65–81)	71 (67–78)	0.315
≥ 75, *n* (%)	3 (21.4)	1 (7.7)	0.596
**Gender**			0.252
Male, *n* (%)	4 (28.6)	7 (53.8)	
**Myeloma subtype, *n* (%)**			0.451
IgG	7 (50.0)	6 (50.0)	
IgA	5 (35.7)	2 (16.7)	
Light chain disease	2 (14.3)	4 (33.3)	
**Light chain type, ***n*** (%)**			0.999
Kappa	9 (64.3)	7 (58.3)	
Lambda	5 (35.7)	5 (41.7)	
**International Staging System stage, n (%)**			0.778
I	6 (42.9)	3 (27.3)	
II	4 (28.6)	4 (36.4)	
III	4 (28.6)	4 (36.4)	
Unknown	0	2	
**Revised International Staging System stage, ***n*** (%)**			0.999
I	3 (21.4)	3 (27.3)	
II	8 (57.1)	6 (54.5)	
III	3 (21.4)	2 (18.2)	
Unknown	0	2	
**Serum lactate dehydrogenase, ***n*** (%)**			0.420
> ULN	6 (42.9)	3 (23.1)	
**Glomerular filtration rate, mL/min/1.73m**^**2**^**, median (range)**	70.93 [22.96, 132.44]	73.04 [28.92, 110.86]	0.923
Creatinine > 2 mg/dL, n (%)	1 (7.1)	0	0.999
**Cytogenetic profile, ***n*** (%)**^**a)**^			0.202
Standard-risk	10 (83.3)	7 (53.8)	
High-risk	2 (18.2)	6 (46.2)	
Unknown	2	0	
**Serum β2-microglobulin, ***n*** (%)**			0.999
≥ 5.5 mg/L	5 (35.7)	4 (36.4)	
Unknown	0	2	
**Serum albumin, ***n*** (%)**			0.999
< 3.5 g/dl	7 (50.0)	6 (46.2)	

*Abbreviations: VMP* bortezomib, melphalan, and prednisone, *Rd* lenalidomide and dexamethasone, *ULN* upper limit of normal

In the Rd group, all patients achieved at least a partial response, compared to 85.7% (12 patients) in the VMP group. Complete responses were reported by 35.7% (5 patients) receiving VMP and 23.1% (3 patients) receiving Rd. The ORR, complete response rate (CRR), and the rate of very good partial response or better (≥ VGPR) did not differ significantly between the two groups (ORR; *p* = 0.481, CRR; *p* = 0.7, ≥ VGPR rate; *p* = 0.999). Within the VMP group, one patient (7.1%) presented with stable disease, and another experienced minimal response. The Rd group displayed no instances of minimal response, stable disease, or progressive disease (Online Resource 2).

After a median follow-up of 30.1 (95% CI: 16.8 months - not reached) and 27.9 (95% CI: 19.6–29.0 months) months for the VMP and Rd groups, respectively, disease progression occurred in 71.4% (10 patients) and 30.8% (4 patients) of the VMP and Rd groups, respectively. The median PFS reached 14.6 months (95% CI: 9.5–16.9 months) for VMP and was not reached for Rd. The log-rank test suggested a trend favoring Rd over VMP in PFS (*p* = 0.053) (Fig. 1A). The hazard ratio for VMP compared to Rd was 3.021 (*p* = 0.064). In the VMP cohort, 28.6% (4 patients) died, while all patients in the Rd group survived. The median OS was not reached in either group, with the log-rank test showing no significant difference in OS between treatments despite a more favorable trend for the Rd group (*p* = 0.054) (Fig. 1B). Subgroup analysis indicated a general trend of better efficacy of Rd over VMP, notably in patients with baseline serum albumin levels lower than 3.5 g/dL, who showed a significantly better PFS with Rd compared to VMP (HR = 8.66, 95% CI: 1.03–72.72, *p* = 0.046) (Fig. 2).

**Fig. 1:**
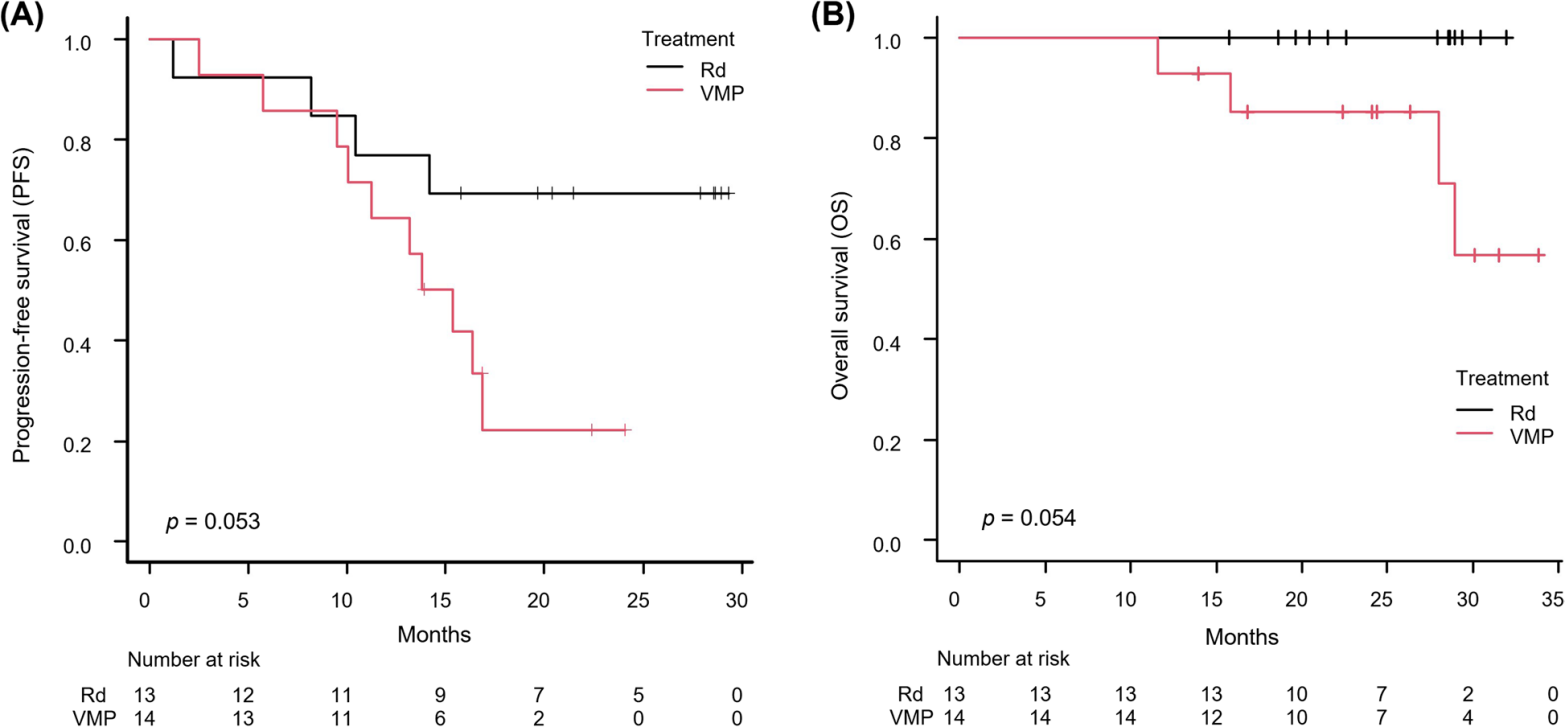
Comparison of survival outcomes between the VMP and Rd groups by Kaplan–Meier method. (**A**) Progression-free survival; (**B**) overall survival. Log-rank tests on both outcomes demonstrated a trend favoring Rd rather than VMP (*p* = 0.053 and 0.054, respectively). Abbreviations: VMP, bortezomib, melphalan, and prednisone; Rd, lenalidomide and dexamethasone

**Fig. 2:**
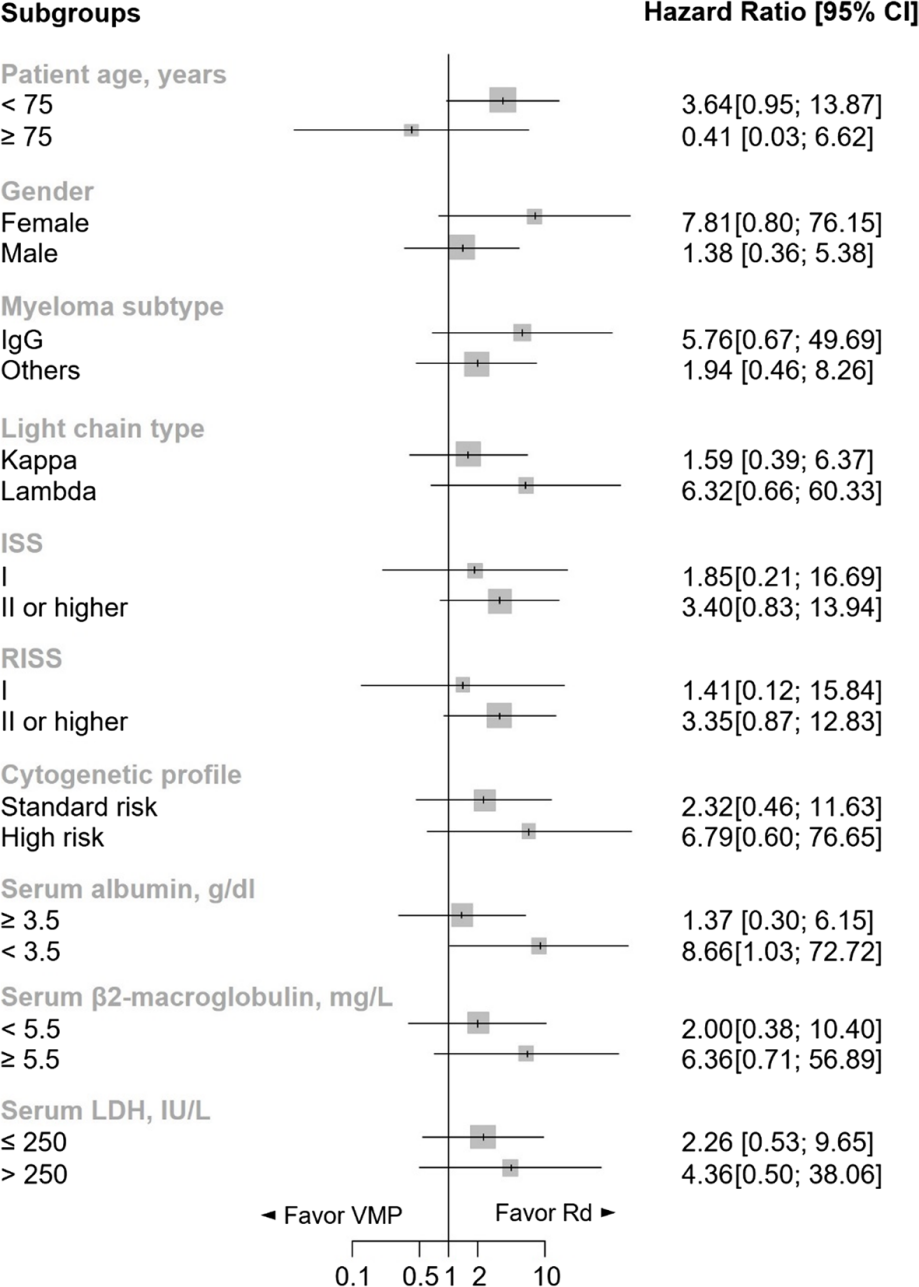
Subgroup analysis of progression-free survival. A hazard ratio > 1 indicates an advantage for Rd. Rd had a trend of better efficacy over VMP in most subgroups. Patients with baseline serum albumin level < 3.5 g/dl showed a significantly better PFS with Rd (hazard ratio, 8.66; *p* = 0.046). Patients aged ≥ 75 years showed a trend of better PFS with VMP (hazard ratio, 0.41; 95% CI: 0.03–6.62). Abbreviations: VMP, bortezomib, melphalan, and prednisone; Rd, lenalidomide and dexamethasone; CI, confidence interval; ISS, International Staging System; RISS, Revised International Staging System; LDH, lactate dehydrogenase

Table 2 shows the prevalent treatment-related adverse events observed. Peripheral neuropathy of any grade was significantly more frequent in the VMP group than in the Rd group (92.7% vs. 41.2%, *p* = 0.033), as were urticaria or rashes (42.9% vs. 0%, *p* = 0.016). Infection of any grade was significantly more common in the Rd group (92.3% vs. 28.6%, *p* = 0.001), with three patients (23.1%) in the Rd group experiencing a grade 3 or 4 infection. Creatinine elevation of any grade occurred in five patients (38.5%) in the Rd group and one patient (7.1%) in the VMP group.

**Table 2:** Adverse Events

Events	Any grade			Grade 3 or 4		
	**VMP**	**Rd**	***p*-value**	**VMP**	**Rd**	***p*-value**
	**(***N*** = 14)**	**(***N*** = 13)**		**(***N*** = 14)**	**(***N*** = 13)**	
**Hematologic, ***n*** (%)**						
Neutropenia	11 (78.6)	12 (92.3)	0.596	7 (50)	7 (41.2)	0.999
Anemia	14 (100)	13 (100)	0.999	1 (7.1)	0	0.999
Thrombocytopenia	1 (7.1)	1 (7.7)	0.999	1 (7.1)	1 (7.7)	0.999
Lymphopenia	11 (78.6)	7 (41.2)	0.236	10 (71.4)	6 (46.2)	0.252
**Non hematologic, ***n*** (%)**						
Fatigue	2 (14.3)	4 (30.8)	0.385	1 (7.1)	0	0.999
Anorexia	2 (14.3)	2 (15.4)	0.999	0	2 (15.4)	0.222
Edema	4 (28.6)	4 (30.8)	0.999	0	0	0.999
Urticaria / Rash	6 (42.9)	0	0.016	1 (7.1)	0	0.999
Constipation	2 (14.3)	6 (46.2)	0.103	0	0	0.999
Diarrhea	1 (7.1)	3 (23.1)	0.326	1 (7.1)	2 (15.4)	0.596
Peripheral sensory neuropathy	13 (92.9)	7 (41.2)	0.033	1 (7.1)	0	0.999
Insomnia	3 (21.4)	3 (23.1)	0.999	0	0	0.999
Infection	4 (28.6)	12 (92.3)	0.001	0	3 (23.1)	0.098
Secondary malignancy	3 (21.4)	3 (23.1)	0.999	3 (21.4)	3 (23.1)	0.999
Liver enzyme elevation	1 (7.1)	1 (7.7)	0.999	0	0	0.999
Creatinine elevation	1 (7.1)	5 (38.5)	0.077	1 (7.1)	3 (23.1)	0.326
Deep vein thrombosis	0	1 (7.7)	0.481	0	0	0.999

*Abbreviations: VMP* bortezomib, melphalan, and prednisone, *Rd* lenalidomide and dexamethasone

Our analysis suggested the potential superiority of Rd over VMP in the management of patients with newly diagnosed transplant-ineligible MM. This inference is supported by recent large-scale RWD (real-world data) analyses. For instance, a retrospective examination of 559 newly diagnosed transplant-ineligible MM patients treated with either VMP (*n* = 443) or Rd (*n* = 116) revealed Rd was superior in terms of ORR, PFS, and OS. [6]. These findings are consistent with the conclusions of a network meta-analysis that endorsed Rd as a preferred comparator for evaluating frontline treatments for transplant-ineligible MM [7] and are further corroborated by a pooled analysis from the GIMEMA-MM-03–05 [8] and EMN01 phase III trials [9], underscoring a PFS advantage for Rd after a median follow-up of 32 months [10].

Our study further delineated the relative efficacy advantage of Rd across most patient subgroups, aligning with both our preliminary observations and the extant RWD analyses. Although the safety profiles of Rd and VMP were comparable, Rd was associated with a higher incidence of infection, highlighting the need for vigilance regarding infection risks when implementing continuous treatment paradigms in MM management.

Nevertheless, the unforeseen premature termination of patient enrollment significantly limited our study and impeded further analysis. The higher proportion of patients with high-risk cytogenetic profiles in the Rd group may also have introduced bias despite the statistical insignificance of the difference. Cautious interpretation is warranted due to these potential biases, and a larger prospective study is required to validate our findings. Furthermore, the omission of frailty scores from the study design hindered our ability to explore frailty-adjusted treatment selection.

In conclusion, our findings suggest that continuous Rd may be a preferred treatment option compared to fixed-schedule VMP in terms of efficacy, with implications for disease management and patient survival. Despite the advent of novel therapeutic combinations such as daratumumab with Rd, daratumumab with VMP, and VRD (bortezomib-lenalidomide-dexamethasone) as standards for newly diagnosed transplant-ineligible MM, the relevance of VMP and Rd persists, especially for frail patients or those contraindicated for more recent regimens. To the best of our knowledge, this is the first clinical trial to juxtapose VMP against Rd directly, offering invaluable insights that might guide decision-making for transplant-ineligible patients with MM precluded from accessing novel treatments.

### Supplementary Information

Supplementary Material 1.

## Data Availability

The data that support the findings of this study are not publicly available due to privacy restrictions. Requests to access the datasets should be directed to the corresponding author.
