# Supplementary material for: Comparative efficacy of VMP vs. Rd in newly diagnosed, autologous stem cell transplant-ineligible multiple myeloma patients: a prematurely terminated randomized controlled study, CAREMM-2002 study
Source: Blood Res. 2024 Jul 17;59(1):24. doi: 10.1007/s44313-024-00025-7 (PMC11254880; doi:10.1007/s44313-024-00025-7)
Supplement: Supplementary file 1 — Supplementary Material 1. [file 44313_2024_25_MOESM1_ESM.docx]

**Comparative Efficacy of VMP vs. Rd in Newly Diagnosed, Autologous Stem Cell Transplant-Ineligible Multiple Myeloma Patients: A Prematurely Terminated Randomized Controlled Study, CAREMM-2002 Study.**

**Running Head:** VMP vs. Rd for transplant-ineligible myeloma

Cheong Yoon Huh^1,#^, Sung-Soo Park^1,#^, Jung Yeon Lee^1^, Chang-Ki Min^1,*^, and the Catholic Research Network for Multiple Myeloma (CAREMM-2002 study)

^1^Department of Hematology, Catholic Hematology Hospital, College of Medicine, The Catholic University of Korea, Seoul, Republic of Korea

**^#^The first author:** C. Y. H., and S.-S.P. contributed equally to this work as first authors.

***Corresponding authors**

**Chang-Ki Min, MD, PhD**

Catholic Hematology Hospital, Seoul St. Mary's Hospital, College of Medicine,

The Catholic University of Korea, 222 Banpodaero, Seocho-gu, Seoul, Korea.

Tel: 82-2-2258-6054 Fax: 82-2-599-3589 E-mail: [ckmin@catholic.ac.kr](mailto:ckmin@catholic.ac.kr)

**Online Resource 1. Endpoints, Evaluations, Statistical analysis**

ORR was defined as the proportion of patients achieving a partial response or better. PFS was measured from the date of randomization to the occurrence of progressive disease or death, whichever came first. Patient evaluations were systemically performed on the first day of each treatment cycle to monitor ongoing responses and side effects. The assessment of treatment response adhered to the International Uniform Response Criteria for Multiple Myeloma, ensuring standardized evaluation across patients. The severity of adverse events was categorized according to the National Cancer Institute Common Terminology Criteria for Adverse Events (NCI CTCAE), version 5.0, offering a detailed framework for evaluating and reporting the safety profile of the therapies.

Categorical variables were analyzed using Fisher’s exact test to ascertain significant differences. For continuous variables, comparisons were made using Student’s t test or Mann-Whitney U test as appropriate depending on the data distribution. Time-to-event outcomes including OS and PFS were evaluated using the log-rank test with Kaplan–Meier method to estimate survival distributions. Prior to leveraging the Cox proportional hazards model for hazard ratio estimation, the assumption of proportional hazards was verified using the Schoenfeld residual test, and then used to estimate hazard ratios. The median follow-up duration was determined via the reverse Kaplan–Meier method. Analysis of PFS and OS were conducted within the intention-to-treat population, encompassing all randomized patients. The evaluation of response rates was restricted to patients assessable for response. Safety data were analyzed for all participants who received at least one dose of a study medication. All statistical analysis were executed using R statistical software, version 4.2.3, with a predetermined significance threshold of *p* < 0.05.

**Online Resource 2. Response Outcomes**

| **Variables** | **VMP** | **Rd** | ***p*-value** |
| --- | --- | --- | --- |
|  | (N=14) | (N=13) |  |
| **Overall response, n (%)** | 12 (85.7) | 13 (100) | 0.481 |
| Complete response | 5 (35.7) | 3 (23.1) | 0.700 |
| ≥ Very good partial response, n (%) | 11 (78.6) | 10 (76.9) | 0.999 |
| Very good partial response, n (%) | 6 (42.9) | 7 (53.8) |  |
| Partial response, n (%) | 1 (7.1) | 3 (23.1) |  |
| **Minimal response, n (%)** | 1 (7.1) | 0 |  |
| **Stable disease, n (%)** | 1 (7.1) | 0 |  |
| **Progressive disease, n (%)** | 0 | 0 |  |

Abbreviations: VMP, bortezomib, melphalan, and prednisone; Rd, lenalidomide and dexamethasone.
